# Neurosurgical treatment of brain tumors in the first 6 months of life: long-term follow-up of a single consecutive institutional series of 30 patients

**DOI:** 10.1007/s00381-015-2792-5

**Published:** 2015-07-15

**Authors:** Tryggve Lundar, Bernt Johan Due-Tønnessen, Arild Egge, Bård Krossnes, Einar Stensvold, Paulina Due-Tønnessen, Petter Brandal

**Affiliations:** Department of Neurosurgery, Oslo University Hospital, Postboks 4950 Nydalen, 0424 Oslo, Norway; Department of Pathology, Oslo University Hospital, Postboks 4950 Nydalen, 0424 Oslo, Norway; Department of Pediatrics, Oslo University Hospital, Postboks 4950 Nydalen, 0424 Oslo, Norway; Department of Radiology, Oslo University Hospital, Postboks 4950 Nydalen, 0424 Oslo, Norway; Department of Oncology, Oslo University Hospital, Postboks 4950 Nydalen, 0424 Oslo, Norway

**Keywords:** Brain tumor in infants, Long-term results, Pediatric neurosurgery

## Abstract

**Object:**

The aim of this study is to delineate the long-term results for patients going through surgery for pediatric brain tumors in the first 6 months of life.

**Methods:**

Thirty consecutive children (1–182 days old) who underwent primary resection for a brain tumor during the years 1973–2012 were included in this retrospective study on surgical morbidity, mortality rate, academic achievement, and/or work participation. Gross motor function and activities of daily life were scored according to the Barthel index.

**Results:**

Of the 30 patients, 11 children had surgery in the first 3 months of life (1 to 88 days) and 19 were aged 3 to 6 months (94–182 days) at the time of surgery. The male/female ratio was 1.0 (15/15). No patients were lost to follow-up. Two patients died in the postoperative period (30 days). Another eight patients died during the follow-up. Twenty patients are alive, with follow-up times from 2 to 38 years, median 13 years. Among the 28 children who survived the primary resection, eight underwent repeat surgery from 6 months to 5 years after the first operation. Two children were operated three times, and one of these also a fourth time. Gross total resection (GTR) was achieved in 20 of the primary resections, subtotal resection (STR) in 6, and in the last 4, only a biopsy or a partial resection was performed.

Nine children received adjuvant chemotherapy and three of these also radiotherapy (in the years 1979–1987). Among the 20 survivors, the Barthel index is normal (100) in 18 patients, 40 in one, and 20 in the last one. Eight tumors were located to the posterior fossa, and 22 were supratentorial. Eighteen tumors were histologically low-grade (WHO grade I–II), most of these were plexus papillomas (7) or astrocytomas (7), and 12 were high-grade (WHO grade III–IV); PNET/medulloblastomas (6), ependymoma (2), glioblastoma (2), teratoma, and plexus carcinoma.

**Conclusion:**

Infants with brain tumors may clearly benefit from surgical resection with favorable results even for prolonged periods of time. Ten children died, two of them with prolonged survival for 9 and 29 years. Among the 20 survivors, a stable very long-term result appears obtainable in 18 also when it comes to quality of life. Four of the survivors have been treated for highly malignant tumors with a follow-up of 5, 11, 14, and 26 years. One of our infant patients treated for GBM in 1982, lived for 29 years, however, with a progressive decline in the quality of life probably due to postoperative whole-brain radiation.

## Introduction

Brain tumors diagnosed in the first 6 months of life are considered to have a very dismal prognosis [2, 3, 13-17, 20]. Surgical brain tumor resection in very young children carries a higher morbidity and mortality risk compared to older children; however, infants may tolerate brain tumor surgery remarkably well. The postoperative survival of such small children with highly malignant tumors was improved in the 1970s and 1980s, when postoperative radiotherapy was implemented [1, 22]. The clinical results over time were, however, worrisome due to the progressive harmful effects of radiotherapy. In most institutions, radiotherapy was therefore deferred in children below the age of 3 years from the late 1980s. Clinical trials with more precise local radiotherapy as well as chemotherapy in small children have been performed for many years, but surgical resection remains a mainstay of the multimodal therapy for most of the youngest brain tumor patients [6–9, 21, 23].

## Methods

### Case identification and data collection

We retrospectively analyzed a consecutive cohort of 30 patients in their first 6 months of life (aged 183 days or younger) who underwent resection for a brain tumor in the Department of Neurosurgery, The National Hospital, Oslo, Norway, between 1973 and 2012. The cases were collected from surgical protocols of the relevant time period, and all cases of intracranial tumors operated in this period were included. Tumor localization was posterior fossa in 8 children (27 %) and supratentorial in 22 children (73 %). Histology has been reviewed by an experienced neuropathologist.

The case record data include sex, age at the time of primary tumor resection, and information on repeat resection as well as management of hydrocephalus.

Scholastic outcome was simplified into normal versus special schooling, and employment attendance into open, sheltered, or no work.

This series comprises our operative experience of brain tumors in infants during four decades. Most of the children (28 out of 30) were operated after introduction of CT in 1977 and MRI in our institution in 1987 (26 out of 30; Table 1). Two patients underwent ventriculography before tumor resection. In the others, the tumor was visualized on preoperative CT/MRI, and repeat MRI scans were introduced in the follow-up program. The aim of the surgical procedure was gross total resection (GTR) or at least a substantial reduction of the tumor volume (STR). The degree of resection was evaluated by the surgeon and after 1987 by immediate postoperative MRI scans, most often in the same anesthetical procedure as the surgery.

**Table 1:** Patient demographics

Patient	Year	Age days	Localization	Histology	Primary resection	Adjuvant therapy	Age at last follow-up or death	Outcome Barthel index
		Sex						
1	1973	F 161	L hemisphere	GBM	STR	No	P.o. death	Dead
2	1975	F 129	Cerebellum	Astrocytoma	GTR	No	38 years	100
3	1979	M 171	Cerebellum	Medulloblastoma	GTR	RT	4 years	Dead
4	1982	F 152	R hemisphere	GBM	GTR	RT	29 years	Dead
5	1987	F 165	R frontal	PNET	GTR	RT, ChT	1.5 years	Dead
6	1987	F 94	Brain stem	Teratoma	STR	No	0.5 years	Dead
7	1987	M 178	Lateral ventr	Plex papillom	GTR	No	27 years	100
8	1988	M 66	Lateral ventr	Plex carcin	GTR	RS, HC	26 years	100
9	1990	F 124	IV ventricle	Medulloblastoma	GTR	No	0.6 years	Dead
10	1990	F 34	Brain stem	Lipoma	Partial res	RSx2	24 years	40
11	1990	M 3	cerebellum	Astrocytoma	GTR	No	23.5 years	100
12	1991	M 18	III ventricle	Plex papilloma	GTR	No	23 years	100
13	1998	M 147	Lateral ventr	Plex papilloma	GTR	No	16 years	100
14	2000	F 46	Lateral ventr	Plex papilloma	GTR	No	14 years	100
15	2000	F 127	L frontal	PNET	GTR	ChT	14 years	100
16	2000	M 147	III ventricle	Plex papilloma	GTR	No	14 years	100
17	2000	F 173	Opt pathway	Astrocytoma	STR	RS	9 years	Dead
18	2002	M 161	Brain stem	Ependymoma	GTR	RS, HC	2 years	Dead
19	2003	M 58	Cerebellum	Epend. blastoma	GTR	ChT, HC	11 years	100
20	2004	M 88	R temporal	Ganglioglioma	GTR	No	10 years	100
21	2004	F 71	Lateral ventr	Plex papilloma	GTR	No	10 years	100
22	2004	M 182	Cerebellum	Medulloblastoma	STR	ChT, RS	1.6 years	Dead
23	2005	F 122	Opt pathway	Astrocytoma	STR	ChT, RS	9.5 years	100
24	2006	F 107	III ventricle	Plex papilloma	GTR	No	8.5 years	100
25	2006	M 56	Opt pathway	Astrocytoma	Biopsy	No	P.o. death	Dead
26	2006	M 1	R hemisphere	SEGA	Biopsy	ChT, HC, RSx2	8 years	100
27	2009	F 164	Opt pathway	Astrocytoma	Partial res	ChT, HC, RSx3	4.5 years	20
28	2009	F 165	R hemisphere	Anaplastic ependymoma	GTR	ChT, RSx2	5 years	100
29	2010	M 142	R hemisphere	Ganglioglioma	GTR	No	4.5 years	100
30	2012	M 48	Hypothalamus	Hamartoma	STR	No	2 years	100

*GTR* gross total resection, *STR* subtotal resection, *RT* radiotherapy, *ChT* chemotherapy, *RS* repeat tumor surgery, *HC* surgery for hydrocephalus, *SEGA* subependymal giant cell astrocytoma, *GBM* glioblastoma, *PNET* primitive neuroectodermal tumor

### Assessment of functional status

The Barthel index score is a well-established and validated scale using ten variables to measure performance in basic activities of daily living (ADL) primarily related to personal care and mobility [18]. Scores range from 0 to 100, and a higher score denotes greater independence. The purpose was to assess functional status and illustrate eventual differences among subgroups within our cohort.

## Results

The age of the children at primary surgery is given in Table 1. Of the 30 patients, 11 children were in the first 3 months of life (1 to 88 days) and 19 were aged 3 to 6 months (94–182 days). The male/female ratio was 1.0 (15/15). No patients were lost to follow-up, which ended by December 31, 2013. Two patients died in the postoperative period (within 30 days from surgery). Another eight patients died during follow-up. Surgery resulted in GTR in 20 patients, STR in 6, partial resection in 2, and in the last two patients, only a biopsy was taken. Twenty patients are alive, with follow-up times ranging from 2 to38 years, median 13 years (Table 1).

The most common clinical presentation in these infants was macrocephaly and symptoms of increased intracranial pressure (tense fontanelle, vomiting), which was seen in 24 cases. These symptoms were particularly pronounced in two infants with acute hemorrhage in the tumor (patients 4 and 15). Five children had one or more seizures preoperatively, and three infants presented with cranial nerve dysfunction.

The histological examination revealed low-grade (WHO grade 1 or 2) tumors in 18 patients (Table 1): seven astrocytomas, seven plexus papillomas, two gangliogliomas, and two hamartomas. Three of the four optical pathway astrocytomas were of the pilomyxoid type (patients 17, 25, and 27 in Table 1). Three of the plexuspapillomas were WHO grade 2 (patients 13, 14, and 24). The two gangliogliomas were of the desmoplastic infantile type (patients 20 and 29).

Twelve tumors were high-grade (WHO grade 3 or 4): six PNET/medulloblastomas, two ependymomas, two glioblastomas (GBM), one teratoma, and one plexus carcinoma.

Two patients died early. One of the postoperative deaths occurred in 1973 when a 5-month-old girl died after a resection of what turned out to be a GBM. The other patient that died postoperatively was a 2-month-old boy with a very large optical pathway astrocytoma. Only a small biopsy was taken. Because of the tumor size the situation was considered palliative and no antineoplastic treatment was indicated. The patient died 2 days postoperatively after the parents had been informed about the situation and consented to stop treatment.

### Adjuvant and further treatment

Repeat resections were performed in eight patients due to tumor progression. Six of eight repeat resections were performed in children with low-grade tumors. Three infants with high-grade tumors (patients 3, 4, and 5) received whole-brain postoperative radiotherapy (45 Gy) in the years 1979–1987; they stayed alive for 4, 29, and 1.5 years, respectively.

Nine patients received chemotherapy, six of the patients with high-grade tumors and three children with low-grade tumors (Table 1). Five patients had CSF shunt procedures due to persistent hydrocephalus.

### Motorical function and activities of daily life

Among the 20 survivors, the Barthel index score is 100 in 18 patients. Patient 27 is alive with disease with a score of 20, but in a terminal condition, after almost continuous treatment for an aggressive optic-pathway glioma including chemotherapy, four tumor resections and several shunt procedures. The last patient (patient 10) presented with tetraplegia and respiratory arrest 34 days old and MRI disclosed a lipoma innside medulla oblongata with exophytic extension to the fourth ventricle as well as behind the cervical medulla. After partial resection, the patient regained spontaneous respiration, swallowing function, and some motorical function in the upper extremities. She underwent repeat resections after 8 and 10 years due to tumor growth and clinical deterioration. At present, she has a Barthell index score of 40, is driving her own car, but is dependent on an electrical wheel chair to move around.

### School, education, and work

Four of the survivors are still below school age (2–5 years), whereas 10 patients are aged 8–16 years and they all follow regular school programs.

Six patients are aged 23–38 years of which three are students or in regular work, one is in full-time sheltered work, and the last two are outside the labor market. One of these (patient 10, see above) is wheel-chair dependent with normal cognition, whereas the other (patient 8) is suffering from severe epilepsy.

### Long-term outcome—surgical resection

There are 20 survivors with follow-up from 2 to 38 years, median 13 years (Table 1). Survival was related to the surgical resection (Fig. 3). Fifteen of the 20 infants who had a GTR at primary surgery are alive with follow-up from 4.5 to 38 years. Five are dead, after 0.6, 1.5, 2, 4, and 29 years. The latter was a 5-month-old girl (patient 4) who underwent GTR for a glioblastoma followed by whole brain radiotherapy (45 Gy) in 1982. Initially, she recovered quite well, but from the age of 12 months she deteriorated over years with severe epilepsy and loss of clinical skills including cognitive functions. There was a continuous negative development until she died 29 years old in status epilepticus. A CT scan showed several radiation induced meningeomas but no evidence of glioblastoma regrowth.

In the ten patients with non-GTR, i.e., STR, partial resection, or biopsy; two patients died in the postoperative period as described above. Among the eight infants that survived the early postoperative period, another 3 are dead after 0.5, 1.6, and 9 years. Five are alive with follow-up periods of 2, 4.5, 8, 9.5, and 24 years.

### Long-term outcome—tumor histology

#### Low-grade

**Fig. 1:**
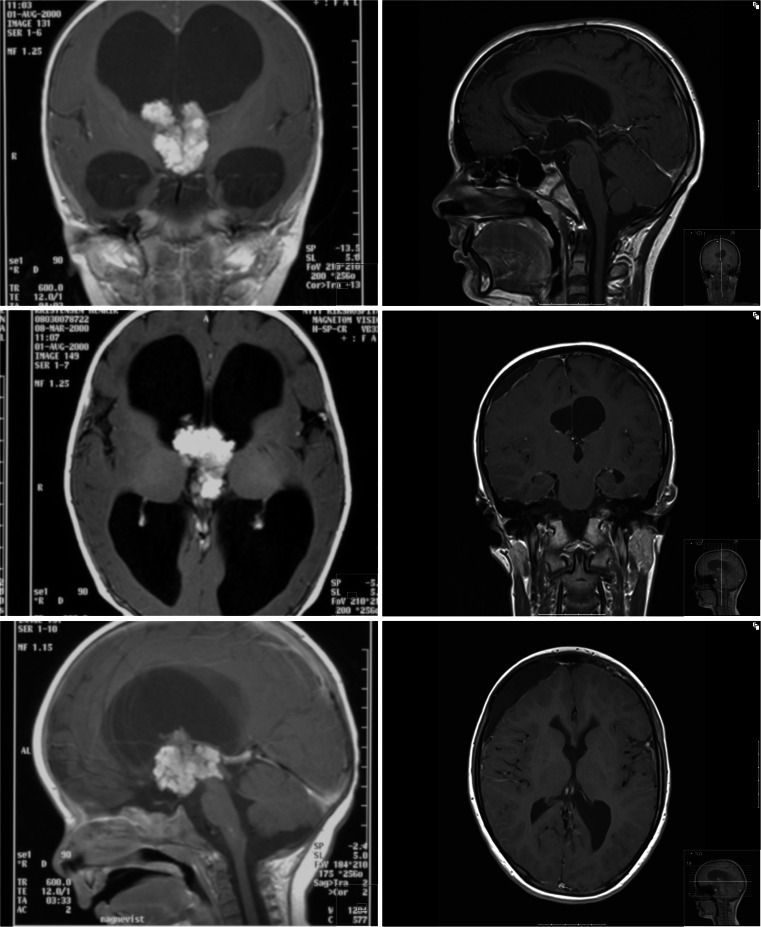
Patient 16. Five-month-old boy presenting with rapidly increasing head circumference and tense fontanelle. Preoperative and postoperative axial and sagittal MRI scans after GTR for the third ventricle plexus papilloma. The patient has 14-year event-free survival

Outcome in terms of survival was clearly related to histology. Sixteen of the 18 infants with low-grade tumors are alive after follow-up periods ranging from 2 to 38 years, median 12 years. Figure 1 shows preoperative and postoperative MRI scans of a 5-month-old boy with a third ventricular plexus papilloma (patient 16). His clinical outcome has been uneventful for the next 14 years. The two deceased patients both had aggressive optical pathway tumors. One died early postoperatively and the other after 9 years and two surgical resections. Figure 4 combines the effect of low-grade histology and GTR versus non-GTR on survival.

#### High-grade

**Fig. 2:**
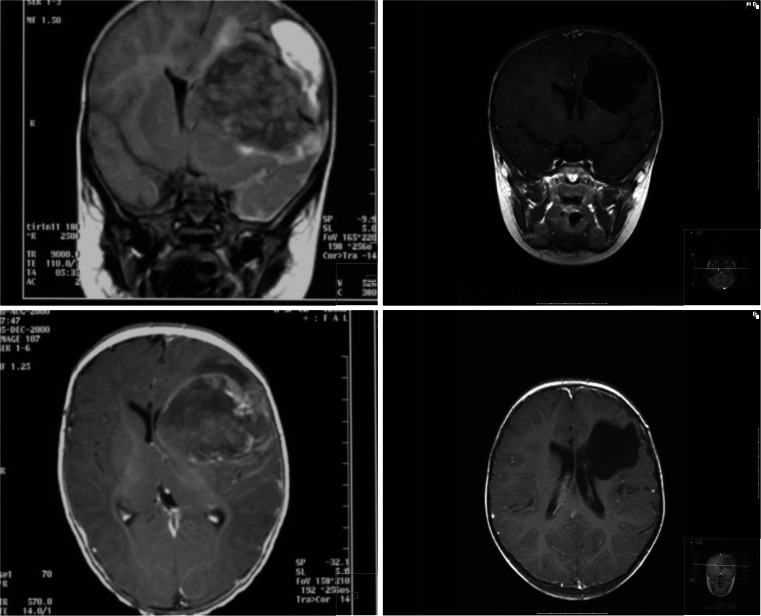
Patient 15. Four-month-old girl with acute symptoms (seizures) due to a hemispheric PNET with acute bleeding. She underwent GTR followed by chemotherapy. She has a 14-year event-free survival. Coronal and axial preoperative and postoperative MRI scans are shown

Twelve patients had high-grade tumors. Four are alive with follow-up periods of 5, 11, 14, and 26 years. One child died in the early postoperative phase; six children died from progressive disease after 0.5, 0.6, 1.5, 1.6, 2, and 4 years in spite of multimodal therapy; and one patient died after 29 years—supposedly from late effects of radiotherapy (see above). Figure 2 shows preoperative and postoperative MRI scans for patient 15. She underwent GTR for a supratentorial PNET followed by chemotherapy according toHIT-2000. Fourteen years later, she follows a normal school program with some assistance, without evidence of recurrent disease.

Two of the children with malignant tumors underwent repeat tumor resection and three had adjuvant chemotherapy after the first tumor resective surgery (Table 1). The clinical condition is stable and the last follow-up MRI did not demonstrate active disease. Figure 4 illustrates that all survivors with malignant disease had a GTR, whereas none of the infants with high-grade neoplasms survived following non-GTR surgery.

## Discussion

Children treated for brain tumors during the first 6 months of life have been considered to have a dismal prognosis, although clinical reports on results for this specific age group are few [16, 24]. Numerous institutional series describing outcome for children following brain tumor surgery in the first year of life have been published [2, 4, 12, 17, 22, 25]. In all these series, the majority of tumors in infants have been supratentorial, a finding corroborated by us. The clinical outcome is described as very challenging for the age group below 6 months of age, both in terms of impaired survival rates and major neurological deficits in many of the survivors [16, 24]. It is well known that age may influence the result. Improved survival figures for children with high-grade brain tumors was the result following the introduction of postoperative radiation therapy, especially in children with medulloblastoma. However, while survival was increased, a reduction in the quality of life was observed over time, more evident in youngest children under the age of 3 years. Our present series also include an example of this pertinent problem—progressive clinical deterioration over 29 years after an uneventful clinical outcome during the first 6 months. The disastrous clinical result, exemplified by this case history, was one of the reasons why we like many others stopped postoperative radiotherapy for high-grade brain tumors in children below the age of 3 years from the late 1980s.

The knowledge about harmful side effects following central nervous system radiotherapy in young age led to several clinical trials of chemotherapy in the management of brain tumors in small children, with the purpose of avoiding or deferring radiation therapy. A number of reports have focused on survival and outcome data for small children treated for high-grade brain tumors with postponed radiation therapy.

Many of these studies underscore the importance of GTR as an individual prognostic factor for survival. Our present series support this opinion, although the absolute numbers are limited. Of the 20 survivors, 14 had undergone a GTR. Additionally, there was a long-term survivor who died after 29 years without signs of progressive tumor disease after GTR for a GBM, although postoperative radiotherapy most probably contributed to this result. All the 11 patients with low-grade tumors who had a GTR are alive, whereas 5 out of 7 with non-GTR are alive, some after repeat resections. Four infants treated for high-grade tumors are alive; all had a GTR. Five others with GTR are dead, all within 4 years from surgery—with the exception of the girl that died after 29 years from treatment side effects, and not from tumor progression. The three children without GTR all died within 1.5 years.

Although GTR seems very important, it should be noted that the favorable survival data from this series may be influenced by several other factors as well. The high incidence of plexus tumors in the first year of life is well known [1, 4, 11, 13, 14, 24, 25], and the case-mix with eight plexus tumors in our series, seven of them papillomas, are tumors with a good prognosis. These patients are all survivors including the infant treated for a carcinoma with surgery alone. The latest follow-up MRIs are without residual tumor. There were also two cases of cerebellar astrocytoma, rather uncommon at this early age and also tumors with generally good long-term results [5]. On the other hand, there were also four low-grade optical pathway astrocytomas, of which two died in spite of aggressive treatment. The disproportionately high incidence of such aggressive tumors in this age group (also called chiasmatic-hypothalamic gliomas) is also well known [22]. Although histologically low-grade, they may be difficult to treat.

The poor prognosis for infants with brain tumors is also related to congenital tumors, and often very large tumors, and aggressive high-grade tumors responding poorly to antineoplastic therapy. In this series, there was no single case of AT/RT among the neonates with PNETs, but one case of ependymoblastoma with an unexpected good long-term survival. Our results suggest that GTR may influence the outcome for these patient groups, but the consequences of radical excision may be additional neurological sequelae. On the other hand, even small children tolerate surgery remarkably well and often recover from severe immediate postoperative neurological deficits with time. Of 28 infants that survived the early postoperative period, seven had repeat surgery and, four of these more than once.

Chemotherapy is a good alternative management strategy for infants with high-grade and also some with low-grade tumors. However, considered the good tolerance also for surgery, further resection should be discussed until further tumor surgery is no longer possible or is inappropriate. Radiotherapy should be deferred as long as possible and high precision techniques should always be used.

The clinical implications of a brain tumor in infancy may be severe for the child and their family. Survival data may illustrate the impact upon prognosis of multimodal treatment with surgery, chemotherapy, and radiotherapy [10, 19]. However, the quality of life for survivors is also very important. Eighteen of 20 survivors in the present series have a good long-term prognosis in terms of prolonged survival. They also have a normal Barthel index score, indicating that they perform activities of daily life unassisted.

Detailed clinical long-term results in previous infant brain tumor cohorts are not available. Apart from three children treated between 1979 and 1987, our infants have not received radiotherapy. The clinical problems they face today are therefore caused by the neoplastic disease, surgical resections and handling of their hydrocephalus (five patients), and chemotherapy. This holds promise for a stable and long-lasting good clinical outcome for these patients, without deterioration over time, like we had to face in the patient who survived whole-brain radiation at this age after GTR for a GBM in 1982.

For infants with brain tumors, a long-term follow-up and in principle life-long follow-up is needed to fully understand the impact of neoplastic disease and its management on quality of life including early school years, higher education, and social integration. As the median follow-up time for our survivors is only 12–13 years, we only know that most of them do fairly well in school. Out of six survivors with 23–38-year follow-up, four are living normal lives as students or in full-time work, whereas two are outside the labor market and with severe medical problems (one with severe epilepsy and, the other wheel-chair dependent but car driver with good cognition).

Some previous series report major disability in most survivors even after resections for low-grade tumors in infants [12, 16, 24, 25]. Our experience is more differentiated with a large number of rewardable outcomes. This may be influenced by the high proportion of plexus tumors and a total proportion of low-grade lesions of 60 %. Our results are more like early reports with a majority of benign lesions leading to good outcome for many of the survivors [1, 4, 22].

**Fig. 3:**
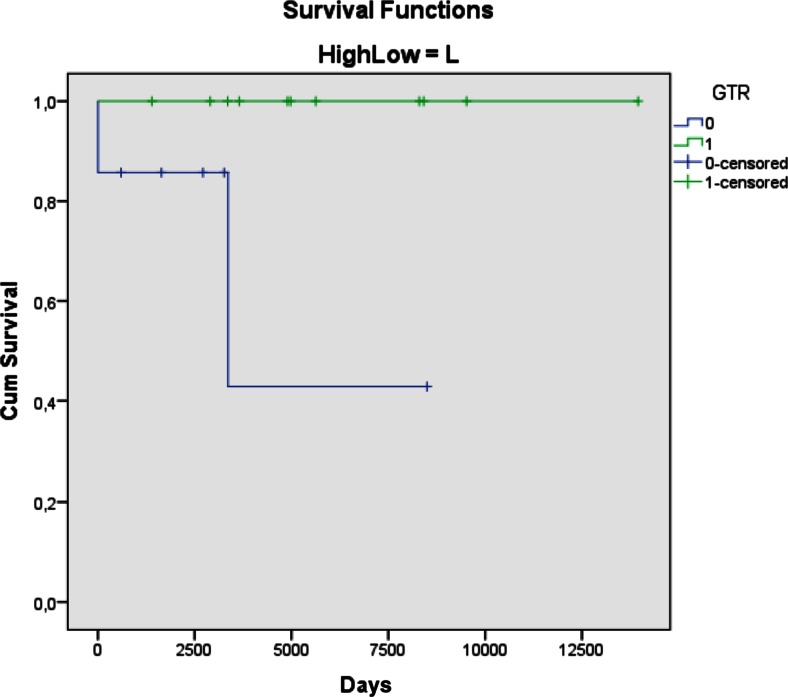
Kaplan-Meier curve presenting survival after GTR (1-green) and non-GTR (0-blue) for low-grade lesions

**Fig. 4:**
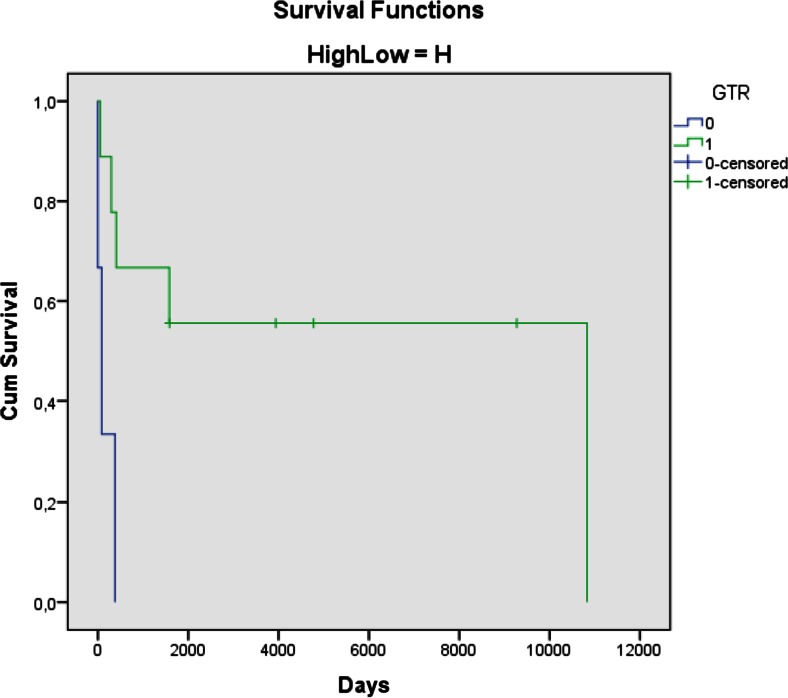
Kaplan-Meier curve presenting survival after GTR (1-green) and non-GTR (0-blue) for high-grade lesions

In our series, no patient has been lost to follow-up (Figs. 3 and 4). Because many of these patients live for many years, radiation of the brain should not be undertaken in low-grade patients, until further resection seems inappropriate. Although the numbers are small for infants with high-grade tumors, GTR followed by chemotherapy appears to give a chance for a relatively good result. The group dying from their malignant disease did so within a few years, at an age where radiotherapy might induce severe harmful effects if they should end up as long-term survivors.

## Conclusion

Brain tumors occurring in the first 6 months of life can be treated with rewarding long-term results in many cases. GTR was achievable in 67 % of infants and led to good tumor control in all children with low-grade lesions and in 4 out of 8 infants with high-grade tumors when followed by chemotherapy.
